# Suicide attempt following sickness absence and disability pension due to common mental disorders: a prospective Swedish twin study

**DOI:** 10.1007/s00127-019-01803-w

**Published:** 2019-11-20

**Authors:** M. Wang, L. Mather, P. Svedberg, E. Mittendorfer-Rutz

**Affiliations:** Division of Insurance Medicine, Department of Clinical Neuroscience, Karolinska Institutet, 171 77 Stockholm, Sweden

**Keywords:** Sick leave, Disability pension, Common mental disorders, Suicide attempt, Twin study

## Abstract

**Purpose:**

The aim of this study was to investigate if sickness absence and disability pension (SA/DP) in general and due to specific common mental disorders (CMDs) are associated with subsequent suicide attempt among women and men by taking familial factors (genetics and shared environment) into consideration.

**Methods:**

This register-based cohort study includes 4871 twin pairs 18–65 years of age discordant for SA/DP due to CMDs 2005–2010. Twins were followed up for suicide attempt from inpatient and specialised outpatient care until December 2012. Conditional Cox proportional hazards regression models, adjusting for familial factors, were used to calculate hazard ratios (HR) with 95% confidence intervals (CI).

**Results:**

SA/DP due to CMDs was associated with a higher risk of suicide attempt (HR 3.14, CI 2.51–3.93). The risk of suicide attempt was five times higher among men and three times higher among women, compared to the SA/DP unaffected co-twins. In the diagnosis-specific analysis, SA/DP due to anxiety disorders resulted in the highest HR (4.09, CI 2.37–7.06) for suicide attempt, followed by depressive disorders (HR 3.70, CI 2.66–5.14) and stress-related disorders (HR 1.96, CI 1.35–2.84). The stratified analysis on zygosity indicates that there seems to be a genetic influence on the associations between SA/DP due to CMDs and suicide attempt, particularly among women and among those with SA/DP due to depressive disorders.

**Conclusions:**

SA/DP due to CMDs was a risk factor for suicide attempt among women and men. Genetic factors might explain part of the associations for women and for those with SA/DP due to depressive disorders.

**Electronic supplementary material:**

The online version of this article (10.1007/s00127-019-01803-w) contains supplementary material, which is available to authorized users.

## Introduction

Suicide attempt is a major global public health challenge and occurs nearly 20 times more frequent than completed suicide [1]. Worldwide, the majority of people who attempted suicide had one or more psychiatric disorders, particularly common mental disorders (CMD), including depressive, anxiety and stress-related mental disorders [2]. The risk of suicide attempt in patients with CMDs is also much higher than in the general population [3]. Moreover, CMDs are often associated with work disability in terms of sickness absence and disability pension (SA/DP) among people of working ages [4–6]. In Sweden, CMDs are one of the main SA/DP diagnostic groups [7, 8]. In 2016, 45% of all sick-leave diagnoses among women and 32% among men were due to CMDs. [9].

Recently, the research interest on SA/DP has increased, since it is considered as a public health issue, leading to prominent policy concerns about large economic costs and possible adverse outcomes for those on SA/DP [4]. SA/DP, as a measure reflecting reduced work ability and with a potential risk of exclusion from the labour market, may have adverse effects such as financial hardship, social isolation and unhealthy behaviour [10, 11]. However, the predominant focus of previous research is on facilitating return to work and risk factors for SA/DP, rather than on consequences of being on SA/DP [12, 13]. To date, few studies have investigated the association between SA/DP and subsequent suicidal behaviour, reporting that the risk of suicide attempt and suicide in individuals on SA/DP due to CMDs is particularly high compared to those without SA/DP [14–17]. Still, the knowledge on the associations between specific SA/DP diagnoses and suicide attempt is limited. Also, using a twin study design is novel and can investigate the role of familial factors (genetic and shared environment, e.g., during childhood) on the associations. Moreover, there is well-documented knowledge regarding sex differences regarding both SA/DP and suicidal behaviour [18, 19]. Generally, women are reported to have higher levels of SA/DP [4, 18] and are more likely to attempt suicide [1] than men, whereas suicide is more common among men [19]. However, sex differences in the association between SA/DP due to CMDs and suicidal behaviour remain unclear.

Also, knowledge is lacking regarding the mechanisms in the association between SA/DP due to CMDs and subsequent suicidal behaviour. As familiar factors are influential on CMDs and suicidal behaviour [20–23] as well as on SA/DP [24–27], an analysis of the contribution of genetics and shared environment to the association between SA/DP due to CMDs and subsequent suicidal behaviour seems important. An advantage of a twin study design is that in matched regression models of discordant twin pairs, it adjusts for many potential unmeasured confounders that the twins are matched on. These include genetics (100% for monozygotic (MZ) and on average 50% for dizygotic (DZ) twin pairs) and common rearing environment (100% for both MZ and DZ twin pairs when reared together), referred to as familial factors [28]. The regression analysis can also be stratified on zygosity and if the estimates are lower in MZ twins that are more closely matched on genetics, compared to DZ twins, we can suspect that genetic factors are of importance for the association [29]. To the best of our knowledge, there is no published study to date on the association between SA/DP due to CMDs and suicide attempt that has considered familial factors.

## Aims

The aim of the study was to investigate the association between all-cause SA/DP and SA/DP due to specific CMDs diagnoses with subsequent suicide attempt among women and men, by taking familial factors (genetics and shared environment) into account.

## Methods

### Sample and data

This study was based on the Swedish Twin project Of Disability pension and Sickness absence (STODS). STODS includes all twins from the Swedish Twin Registry (STR) born between 1925 and 1990, i.e., 119 907 twin individuals. Extensive survey data were collected for these individuals, including information on zygosity. The zygosity diagnosis was confirmed using DNA markers in a subset of the sample and proved correct in 98% of the pairs. Register data were used from 1st January, 2001 up to 31st December, 2012 from:

1. Statistics Sweden’s Longitudinal Integration Database for Health Insurance and Labour Market Studies (LISA) that includes age, sex and emigration.
2. The Social Insurance Agency’s MicroData for Analysis of the Social Insurance database (MiDAS) that contains dates for SA and DP from 1994 and diagnoses from 2005 and onwards.
3. The National Board of Health and Welfare’s National Patient Register that contains date and cause of in and specialised outpatient care starting from 1973 and from 2001, respectively as well as the cause of death register that contains information on date and cause of death from 1961 and onwards.

**Fig. 1:**
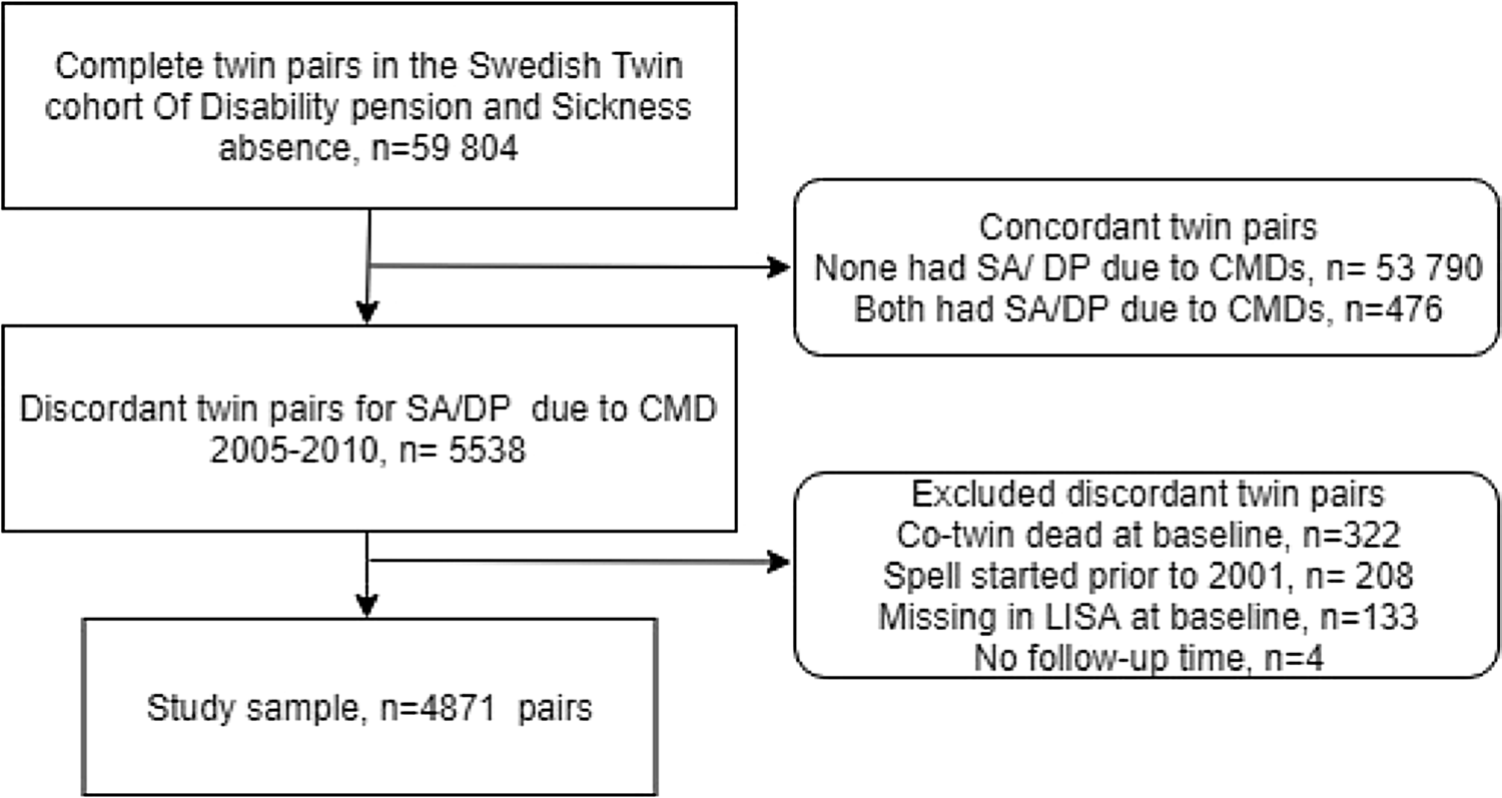
Flow chart for the study population. *SA/DP* sickness absence and disability pension, *CMDs* common mental disorders, *LISA* longitudinal integration database for health insurance and labour market studies

SA and DP was measured from the start of a sick-leave spell and start date of the disability pension granting (cohort entry date), respectively. The inclusion period for both exposure measures was from 2005 to 2010. Both ongoing and new spells were considered, regardless of sick-leave length. The ongoing spells were included if they started 2001 or after, since we had access to all patients’ register data from this point. If an individual had both SA and DP during the inclusion period, the chronologically first event was used and the first day in the SA or DP spell was defined as the start of the follow up. Co-twins who had died at baseline (cohort entry date), had SA/DP that started prior to 2001, were missing in LISA at baseline or had no follow-up time, were excluded. This left a final sample of all twin pairs discordant for SA/DP due to CMDs where both twins were alive, living in Sweden and one of the twins in a pair started SA/DP due to CMDs at the earliest 2001, *n* = 4871 pairs 18–65 years of age (Fig. 1).

### Exposure

Exposure was categorised into SA or DP due to CMDs, other SA/DP diagnoses or no SA/DP. All SA/DP diagnoses were defined by the corresponding codes of the International Classification of Diseases (ICD) version 10 (ICD-10). CMDs were grouped into depressive disorders (ICD-10 F32, F33), anxiety disorders (ICD-10 F40-42) and stress-related disorders (ICD-10 F43). Information on SA spells shorter than 14 days (for employed individuals) was not available, and therefore not included in the analysis.

### Outcome

Suicide attempt from inpatient and specialised outpatient care was defined based on ICD-10 codes X60-84 and Y10-34 and the date of the first of such an event was used.

### Statistical analysis

Kaplan–Meier estimates were applied to describe proportions of suicide attempts during the follow-up time following SA/DP due to CMDs (Supplementary figure). Conditional Cox proportional hazards regression models were used to calculate hazard ratios (HR) with 95% confidence intervals (CI). Follow-up time started at the cohort entry date and was censored for date of death, emigration (missing for two consecutive years from the LISA register), suicide attempt or end of follow-up, i.e., 31st of December 2012, whichever came first. A *p* value < 0.05 was considered statistically significant and the global and graphic tests were used to determine if hazards were proportional. Analyses were stratified by sex and zygosity and CMD diagnosis. Two sensitivity analyses were also performed, one where those with previous suicide attempts (2001–2012) were excluded and one where the pairs where the co-twin had SA/DP in another diagnosis than CMDs during the study period were excluded. STATA IC13 was used to analyse data.

### The Swedish social insurance system

All Swedish residents aged 16 or older with income from work or unemployment benefits can be granted SA benefits in case of reduced work capacity due to disease or injury [30]. For employees, this is paid by the employer during the first 14 days and by the Social Insurance Agency afterwards. All other groups have sickness benefits from Social Insurance Agency. All have a qualifying day without any benefits while self-employed individuals may have more qualifying days. All residents aged 19–65 can be granted DP, if their work capacity is long term or permanently reduced due to disease or injury. The SA benefits cover approximately 80%, while DP covers up to 65% of the income. Both SA and DP can be granted for full or part-time (25%, 50% or 75%) of ordinary work hours. The Swedish social insurance system is comparable to systems in the Nordic countries, but is more generous compared to many other countries, for example, the US. In Sweden the whole population is covered by the system and individuals can receive reasonably higher benefits with lower entry thresholds and better rehabilitation measures compared to individuals in the US [31].

### Ethics approval

The study was approved by the Regional Ethical Review Board of Stockholm, Sweden and has therefore been performed in accordance with the ethical standards laid down in the 1964 Declaration of Helsinki and its later amendments.

## Results

### Descriptive analyses

Table 1 contains information on frequencies of the outcome and covariates stratified by the exposure. In the whole sample that had suicide attempt, the average time from the index sick leave and suicide attempt was 2.5 years. For different subgroups, the time was similar for women and men (2.4 years for women and 2.8 years for men). There was some difference in regards to previous suicide attempt; for those that had a previous suicide attempt it was 1.7 years and for those that did not it was 2.7 years. The average follow-up time was 6.1 years. Hazards were proportional according to the global test, *p* = 0.0518. The mean age in the sample was 44 years (standard deviation was 11.9). Among the twins with SA/DP due to CMDs, 3414 (70.1%) were women, the corresponding number for the co-twins was 2575 (52.9%). There was a larger proportion of individuals with SA/DP due to CMDs who had stress-related disorders (2266, 46.5%), followed by depressive disorders (1925, 39.5%) and anxiety disorders (680, 14.0%). The prevalence of suicide attempt during follow up in the sample was 2.8% (*n* = 272). Moreover, among the twins with SA/DP due to CMDs there was a higher proportion of individuals with previous suicide attempt (2.4% vs. 1.3%) and suicide attempt during follow-up time (4.2% vs. 1.4%) compared to their co-twins. The results from the Kaplan–Meier analyses suggested that the 5-year suicide attempt probability was higher in twins with SA/DP due to CMDs than the co-twins (Supplementary figure).

**Table 1:** Frequencies among the 4871 discordant twin pairs

	Twins with SA/DP due to CMDs = 4871	Co-twins without SA/DP due to CMDs = 4871
Sex		
Men	1457 (29.9)	2296 (47.1)
Women	3414 (70.1)	2575 (52.9)
Zygosity		
Monozygotic	1101 (22.6)	1101 (22.6)
Dizygotic same sex	1315 (27.0)	1315 (27.0)
Opposite sex	1771 (36.4)	1771 (36.4)
Unknown zygosity	684 (14.1)	684 (14.1)
Mean age at the cohort entry date (18–65 years)	43.6 (SD 11.9)	43.6 (SD 11.9)
SA/DP^a^		
CMDs^b^	4871 (100)	
Other diagnoses of SA/DP		2008 (41.2)
No SA/DP		2863 (58.8)
CMD SA/DP diagnosis		
None		4871 (100)
Depressive disorders	1925 (39.5)	
Anxiety disorders	680 (14.0)	
Stress-related disorders	2266 (46.5)	
Previous suicide attempt^c^		
Yes	118 (2.4)	65 (1.3)
No	4753 (97.6)	4806 (98.7)
Death during the follow up		
Suicide^d^	25 (0.5)	5 (0.1)
Other cause of death	100 (2.1)	148 (3.0)
Alive	4746 (97.4)	4718 (96.9)
Outcome suicide attempt		
Yes	203 (4.2)	69 (1.4)
No	4668 (95.8)	4802 (98.6)

^a^SA/DP: sickness absence and disability pension

^b^CMDs: common mental disorders

^c^Previous suicide attempt was measured based on ICD-10 codes X60-84 and Y10-34 in 2001–2012

^d^Suicide was measured based on ICD-10 codes X60-84 and Y10-34

### Suicide attempt

In the whole sample, individuals with SA/DP due to CMDs had a significantly higher risk of subsequent suicide attempt than their co-twins (HR 3.14, 95% CI 2.51–3.93). This high risk of suicide attempt remained after stratifying for sex. For men with SA/DP due to CMDs the risk of suicide attempt was fivefold higher while for women the risk was nearly threefold higher than their co-twins. In the analysis with MZ twins, the HR was lower compared to DZ twin pairs in the whole sample and in women, which indicates that genetic factors may be involved in the association (Table 2).

**Table 2:** Hazard ratios for suicide attempt among twins discordant for SA/DP due to CMDs

	Conditional Cox, all	Opposite sex DZ	Same sex DZ	MZ
All	3.14 (2.51–3.93)	3.09 (2.11–4.53)	4.09 (2.37–7.06)	2.82 (1.83–4.36)
Men	5.10 (2.86–9.09)		8.00 (2.14–29.88)	8.00 (2.14–29.88)
Women	2.56 (1.87–3.51)		3.22 (1.77–5.86)	2.13 (1.34–3.38)

*SA/DP* sickness absence and disability pension, *CMDs* common mental disorders, *DZ* dizygotic twin pairs, MZ monozygotic twin pairs

In the stratified analysis on specific CMD SA/DP diagnoses, all disorders showed a higher risk of suicide attempt, particularly for anxiety disorders (HR 4.09, 95% CI 2.37–7.06) and depressive disorders (HR 3.70, 95% CI 2.66–5.14). Due to the wide and overlapped CIs, there were no significant differences between different SA/DP diagnoses. The HR was attenuated in MZ twin pairs who had SA/DP due to depressive disorders compared to DZ twin pairs. Therefore, the association between SA/DP due to depressive disorders seems to be influenced by genetic factors (Table 3).

**Table 3:** Hazard ratios for suicide attempt among twins discordant for SA/DP due to CMDs, stratified on type of disorders

	Conditional Cox, all	Opposite sex DZ	Same sex DZ	MZ
Depressive disorders	3.70 (2.66–5.14)	4.00 (2.19–7.31)	5.50 (2.60–11.62)	2.30 (1.31–4.04)
Anxiety disorders	4.09 (2.37–7.06)	3.20 (1.44–7.13)	5.00 (0.81–31.01)	5.50 (1.50–20.10)
Stress-related disorders	1.96 (1.35–2.84)	2.00 (1.06–3.77)	1.75 (0.71–4.31)	2.80 (1.26–6.23)

*SA/DP* sickness absence and disability pension, *CMDs* common mental disorders, *DZ* dizygotic twin pairs, MZ monozygotic twin pairs

### Sensitivity analyses

Table 4 shows HRs for suicide attempt among twins discordant for SA/DP due to CMDs after excluding individuals with previous suicide attempt. There was a significant association between SA/DP due to CMDs and suicide attempt in the whole sample as well as in men and women. Compared to the sample, including information on previous suicide attempts, the HRs were similar. In the analysis of excluding other SA/DP diagnoses than CMDs from the reference group, there was a higher suicide attempt risk for those with SA/DP due to CMDs in the whole sample and in the sex-stratified analysis (Table 5). Compared to the sample with other SA/DP diagnoses in the reference group, the risk estimates were somewhat higher, but the differences were not significant.

**Table 4:** Hazard ratios for suicide attempt among twins discordant for SA/DP due to CMDs, with those with previous suicide attempt excluded

	Conditional Cox, all	Opposite sex DZ	Same sex DZ	MZ
All	3.23 (2.49–4.18)	3.11 (2.04–4.75)	4.13 (2.18–7.82)	3.08 (1.84–5.17)
Men	4.22 (2.31–7.72)		6.00 (1.63–22.05)	6.50 (1.76–24.00)
Women	2.90 (1.97–4.29)		3.50 (1.68–7.29)	2.40 (1.37–4.22)

*SA/DP* sickness absence and disability pension, *CMDs* common mental disorders, *DZ* dizygotic twin pairs, MZ monozygotic twin pairs

**Table 5:** Hazard ratios for suicide attempt among twins discordant for SA/DP due to CMDs, with those with other SA/DP diagnoses than CMDs removed from the reference group

	Conditional cox all	Opposite sex DZ	Same sex DZ^c^	MZ
All	4.86 (3.26–7.23)	3.45 (2.01–5.94)	10.00 (2.65–37.75)	4.00 (1.78–8.98)
Men	15.00 (3.91–57.58)		Too few	Too few
Women	4.25 (2.24–8.06)		5.00 (1.38–18.18)	2.60 (1.17–5.78)

*SA/DP* sickness absence and disability pension, *CMDs* common mental disorders, *DZ* dizygotic twin pairs, MZ monozygotic twin pairs

## Discussion

The current study, based on a prospective cohort of 4871 discordant twin pairs, shows that SA/DP due to CMDs predicts subsequent suicide attempt among women and men even after adjustment for familial factors. Specific CMD SA/DP diagnoses also showed higher risks of suicide attempt. Moreover, we found a possible genetic influence on the association between SA/DP due to CMDs and suicide attempt in the whole sample and among women as well as among those with SA/DP due to depressive disorders.

Previous findings showed that SA and DP due to mental disorders, particularly CMDs were associated with a 2–3 times higher risk of suicide attempt in the general population [32–34]. In this study, we further investigated this relation and found an association between SA/DP due to CMDs and suicide attempt, indicating a threefold higher HR even after adjusting for familial factors by matching. As SA/DP is a complex phenomenon and may be influenced by various factors, it is a challenge to study the effect from SA/DP per se and from the underlying disorders. Using a twin study design provides the benefit of adjusting for many unmeasured confounders in terms of genetics and early rearing environment in studying the association between SA/DP due to CMDs and suicide attempt. In this study, we also observed that the risk for suicide attempt in MZ twins was reduced to some extent. Due to the fact that MZ twins share 100% of their genes and DZ twins share on average 50% of their genes, the lower HRs in MZ twins compared to DZ twins might be due to the more closely matched genetics in MZ twins than DZ twins. Thus, this may indicate a genetic susceptibility to the underlying disorders that lead to SA/DP and may explain the higher risk of subsequent suicide attempt. This finding is also in accordance with previous studies, showing that SA and DP due to mental disorders are influenced by genetic factors [26, 35]. Moreover, the lower risk of suicide attempt in MZ twins compared to DZ twins could also be explained by the fact that MZ twins included in the study had less severe CMDs than the DZ twins, since the more severe CMDs have higher heritability and only discordant pairs were included. This indicates that the same genetic factors may predispose an individual to future suicide attempt and SA/DP due to more severe CMDs, as the HRs were lower in DZ twins. However, even though our results indicate that genetic factors do influence the associations, we found an almost threefold risk within MZ twin pairs indicating there is a direct association between SA/DP due to CMD and suicide attempt.

A high risk of suicide attempt was found among both women and men with SA/DP due to CMDs, but we did not observe any significant sex differences on the association studied. This finding is not in line with a previous study, which has reported that among those with SA due to mental disorders, men had a higher risk of suicide attempt (3.64-fold higher risk) than women who had a 2.98-fold higher risk after adjustment for socio-demographic factors, previous mental healthcare and prescription of psychiatric medication as well as previous suicide attempt [14]. This previous study was based on a larger sample than ours, which may facilitate the detection of significant sex differences. However, we did find a possible genetic influence in women, rather than men for the risk of suicide attempt after SA/DP due to CMDs. Generally, women are more likely to attempt suicide and to be granted SA/DP compared to men [1, 4]. Our result might reveal a familial predisposition to a CMD that is associated with the higher risk of suicide attempt in women. For men, it seems that other factors than familial ones influence the risk of suicide attempt after SA/DP. In fact, men have a higher threshold for reporting health problems and for help-seeking than women [36]. Compared to women, men also may be more likely to have unfavourable health-related behaviours such as high alcohol consumption, which might result in an elevated risk of SA/DP and suicide attempt [37].

Furthermore, our results are in line with previously reported findings that specific CMD SA/DP diagnosis lead to a higher risk of suicide attempt, that is, research showing that DP due to depressive, anxiety and stress-related disorders was associated with a 3–16 times higher risk of suicide attempt in young adults [33, 34]. Again, we also found that the high risk of suicide attempt was likely to be influenced by genetic factors among those with SA/DP due to depressive disorders. The genetic influence for depressive disorders is recognised in previous research [22] and our results indicate that the same genetics also influence suicide attempt. Since, we found that genetics did not seem to affect the associations for those with SA/DP due to anxiety disorders and stress-related disorders, other factors might be involved in the association such as poor work environment, high job strain and low social support [35, 38]. Hence, our results point to that there may be different paths to suicide attempt depending on the underlying disorder of the SA/DP. For depression, the path seems to be by-genetically influenced severity of disease and for stress-related and anxiety disorders environmental factors.

### Methodological considerations

Strengths of this study were the use of Swedish nationwide register data with high quality [39, 40], which minimised loss to follow up and recall bias. Data on SA/DP might include misclassifications and under reporting of CMDs as well as missing data on SA/DP diagnoses [30]. However, a study showed acceptable validity of sick-leave diagnoses [41]. Previous studies have also shown comparable results in SA/DP due to mental disorders between the twin cohort and the general population [42]. Moreover, we were able to include a large sample of discordant twin pairs, which allowed us to analyse rare outcomes such as suicide attempt. However, some analyses still lacked power and CIs were broad. When utilising the co-twin design, there is a risk that we introduce bias by selecting only the discordant pairs, if the majority of the pairs are concordant. However, in the current study, the majority of the pairs were discordant (see, Fig. 1) [43]. The findings in this study can be generalised to working-aged individuals living in countries with comparable economic and labour market situations and health care and social insurance systems.

Other limitations include that we only considered suicide attempts, which require inpatient and specialised outpatient care. We might have missed medically less serious suicide attempts, for example suicide attempts from primary care and suicide attempters who did not require treatment in healthcare, which were not possible to capture in the current register. Furthermore, sick-leave spells < 14 days were not included for employed individuals. It is also important to bear in mind that we only have information on the main diagnosis of SA and was not able to analyse SA due to CMDs as a side diagnosis. Moreover, the current study design does not allow drawing any conclusions on mechanisms and causal relations.

## Conclusions

To conclude, SA/DP due to CMDs was associated with subsequent suicide attempt among women and men after controlling for familial factors. We found a higher risk for suicide attempt following SA/DP due to specific CMDs, particularly for anxiety and depressive disorders. Genetic factors seem to explain part of the associations, especially for women and for those with SA/DP due to depressive disorders.

## Electronic supplementary material

Below is the link to the electronic supplementary material.

Supplementary material 1 (TIFF 732 kb)
