# Supplementary figures and images for: Suicide attempt following sickness absence and disability pension due to common mental disorders: a prospective Swedish twin study
Source: Soc Psychiatry Psychiatr Epidemiol. 2019 Nov 20;55(8):1053–60. doi: 10.1007/s00127-019-01803-w (PMC7395035; doi:10.1007/s00127-019-01803-w)

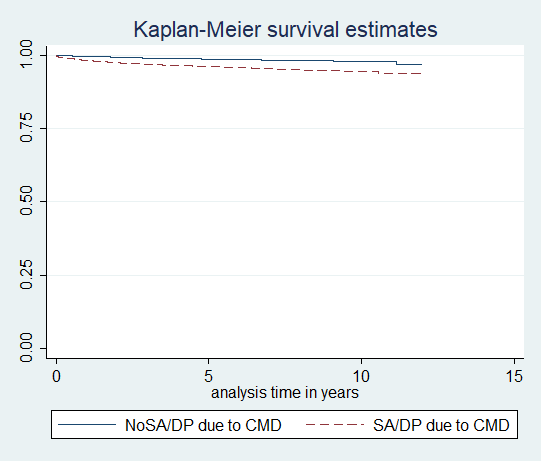

Supplement: Supplementary file 1 — Supplementary material 1 (TIFF 732 kb) [file 127_2019_1803_MOESM1_ESM.tif]
